# Preparation and characterization of monodisperse molecularly imprinted polymer microspheres by precipitation polymerization for kaempferol

**DOI:** 10.1080/15685551.2016.1239174

**Published:** 2016-10-21

**Authors:** Qiang Xia, Yanbin Yun, Qiang Li, Zejun Huang, Zhixia Liang

**Affiliations:** ^a^ School of Environmental Science and Engineering, Beijing Forestry University, Beijing, China; ^b^ School of Science, Beijing Forestry University, Beijing, China

**Keywords:** Molecularly imprinted polymer, microspheres, selectivity, kaempferol, precipitation polymerization

## Abstract

A new kind of molecularly imprinted polymer (MIP) microspheres for the selective extraction of kaempferol was prepared by precipitation polymerization using 4-vinylpridine (4-VP) and ethylene glycol dimethacrylate (EDMA) as functional monomer and cross-linker respectively. The synthesis conditions, such as ratios of 4-VP/EDMA and polymerization time were discussed in detail. Results showed that the 2% was the optimal concentration of co-monomers to obtain monodisperse MIP microspheres, the best ratio of 4-VP/EDMA was 1:2, and 24 h was considered as the proper polymerization time. Compared with the MIP agglomeration or coagulum particles, monodisperse MIP microspheres showed the better adsorption capacity: the saturated adsorption capacity of monodisperse MIP microspheres was 7.47 mg g^−1^, the adsorption equilibrium could be obtained in 30 min. Finally, the adsorption performances of the optimal MIP microspheres were evaluated by kinetic adsorption, adsorption isotherm, and selective adsorption experiments, which indicated that the adsorption mechanism were chemical single layer adsorption and the separation factor was up to 3.91 by comparing with the structure similar compound (quercetin). The MIP microspheres exhibit prospects in the kaempferol efficient and selective separation.

## 1. Introduction

Kaempferol is a natural flavonoid which contains various biological functions, such as antidepressant property, powerful antioxidant activity, anti-inflammatory, increasing metabolism, and cancer fighting properties.[1,2] It is significant to separate kaempferol from flavonoids, and there are already some traditional technologies have been applied in selective extraction of kaempferol, including high performance liquid chromatography,[3] macro-porous resin,[4] and supercritical fluid.[5] But, because of low concentration of kaempferol in nature plant, the complexity of samples, and the structural similarity to other flavonoids, traditional technologies for separating kaempferol have some defects, such as poor separation efficiency, high energy consumption. Therefore, new and effective method needs to be developed to improve the efficiency of kaempferol separation from flavonoids.

Molecularly imprinting is a versatile and facile technique to prepare tailor-made polymers with highly selectivity towards a given target molecule by co-polymerising suitable functional monomers and cross-linkers in the presence of template; when the template molecule is removed, specific cavities and binding sites will be formed within the rigid polymer, which has a high binding affinity and selectivity towards the template molecule.[6–8] The obtained molecularly imprinted polymer (MIP) hold many advantages when compared with nature receptors, such as high selectivity, stability, mechanical, and thermal stability, low cost, and wide range of operating conditions. Due to the unique properties and advantages of the MIPs, they have received great development in various fields, including stationary phases for high-performance chromatography,[9] catalysis,[10] chemosensor technology,[11] and adsorbent for solid phase extraction.[12] In recent years, several MIPs for the separation of kaempferol have been prepared.[13,14] However, most of MIPs for kaempferol were synthesized by bulk polymerization, followed by a grinding and sieving process to obtain the desired particles, which process resulted in irregularly shaped materials with heterogeneous size and porosity. Such particles possess lower the specific surface area and many imprinting sites would be destroyed in grinding processes, which lead to a lower adsorption capacity to kaempferol. In addition, the irregularly particles are not well suited as packing materials for resin or HPLC, so the application of these MIPs in kaempferol separation is difﬁcult. To overcome these drawbacks and optimize the performance of MIP particles, alternative synthetic strategies to monodisperse MIP microspheres that obviate the need for grinding and sieving have evolved, including seeded polymerization,[15] suspension polymerization,[16] emulsion polymerization.[17] These methods have undoubted value, however, residual stabilizers or emulsifiers are adsorbed on the microspheres’ surface potentially affecting the selective and adsorption capacity of MIP to template molecules, the general applicability is questionable in some cases.

Precipitation polymerization was a simple and general method for producing high-quality, monodisperse MIP microspheres without any stabilizers or emulsifiers. Microspheres were formed predominantly by entropic precipitation of primary particles, and followed by continuous capture of oligomers from solution.[18] High-quality MIP microspheres have been obtained with typically uniform size by precipitation polymerization, which have then been used in separation techniques and analytical techniques.[19,20] In precipitation polymerization, the right choice of cross-linker and concentration of co-monomers (the sum concentration of functional monomer and cross-linker) are of utmost importance to form monodisperse spherical particles. Up to now, divinylbenzene (DVB) has been the most common cross-linker in precipitation polymerization, and DVB as cross-linker, only the co-monomers’ concentration was less than <5% (w/v), can monodisperse MIP microspheres be yielded.[21] But if other cross-linker as co-monomer, how to control the critical concentration to form monodisperse MIP microspheres may be different. EDMA was a frequently used cross-linker with high cross linking degree in polymerization reaction. Because the molecular chain of EDMA was longer than DVB, EDMA can form bigger imprinted cavities in the polymer. It can be concluded that the EDMA was a more suitable cross-linker for big template molecule, like kaempferol. As there is a growing need for the direct production of MIP microspheres, the investigation of that using EDMA as cross-linker to prepare MIP microspheres by precipitation polymerization is significant. And the results in this study have demonstrated that monodisperse MIP microspheres with EDMA as cross-linker can be formed by controlling polymerization conditions by precipitation polymerization.

In this paper, monodisperse MIP microspheres were synthesized by precipitation polymerization using EDMA as cross-linker, 4-vinylpridine (4-VP) as functional monomer for selective adsorption of kaempferol. To obtain the best polymerization conditions, the effect of co-monomers' concentration, ratios of 4-VP and EDMA, and polymerization time on MIPs’ morphology and adsorption capacity were investigated. The kinetic adsorption and adsorption isotherm were used to study adsorption mechanism of the MIP microspheres. Quercetin (similar with kaempferol) was selected as a competitive compound to analyze the selectivity of the MIP microspheres.

## 2. Experimental

### 2.1. Materials and methods

Kaempferol (≥98%) and quercetin (≥98%) were obtained from Xi’an Tonking Biotech Co., Ltd, Shanxi, China; Ethylene glycol dimethacrylate (EDMA), 4-VP were purchased from Alfa Aesar, EDMA and 4-VP were purified by reduced pressure distillation to remove inhibitor before polymerization. 2, 2-azobisisobutyronitrile (AIBN) was purchased Tianjin Jinke fine chemical institute, Tianjin, China. Acetonitrile, methanol (MeOH), ethanol (EtOH) and acetic acid were all analytical grade and purchased from Beijing Chemical works, Beijing, China.

### 2.2. Preparation of MIP microspheres

A series of kaempferol MIP microspheres with different polymerization conditions (shown in Table 1) were prepared by precipitation polymerization using EDMA as cross-linker and 4-VP as the functional monomer in acetonitrile. In a typical synthesis, the template molecule kaempferol (0.50 mmol) was dissolved in acetonitrile (62.2 ml), then the solution was degassed in an ultrasonic bath for 5 min and sparged with oxygen-free nitrogen for 10 min. After that, the functional monomer 4-VP (5.0 mmol), cross-linker EDMA (10.0 mmol) and initiator AIBN (50.0 mg) were added to the solution and were stirred for 0.5 h at 300 rpm at room temperature to fully dispersed kaempferol, 4-VP and EDMA. The temperature of the final mixture was increased from room temperature to 80 °C over 0.5 h and was polymerized at 80 °C oil bath under a nitrogen atmosphere with gently stirred for 24 h. At the end of the reaction, the polymers were separated from the reaction medium by filtration on a membrane filter. Following this, the polymers were extracted with a mixture of MeOH/acetic acid (9:1, v/v) in soxhlex extractor to remove kaempferol until no kaempferol was detected by UV spectrophotometer in extraction solutions and washed with MeOH for 24 h. Then, the wet polymers were dried at 60 °C in a vacuum oven for 24 h.

**Table 1.** Preparation and characterization of MIP microspheres.

Sample	Concentration (w/v)	Ratios	Time (h)	Particle morphology	
MIP1	1%	1:2	24	Microspheres	Monodisperse
MIP2	2%	1:2	24	Microspheres	Monodisperse
MIP3	4%	1:2	24	Microspheres	Agglomeration
MIP4	6%	1:2	24	Microspheres	Agglomeration
MIP5	8%	1:2	24	Coagulum	–
MIP6	2%	1:1	24	Microspheres	Agglomeration
MIP7	2%	2:1	24	Coagulum	–
MIP8	2%	1:4	24	Microspheres	Monodisperse
MIP8	4%	1:2	12	Microspheres	Agglomeration
MIP9	4%	1:2	6	Microspheres	Agglomeration

Notes: Reaction conditions: Acetonitrile solvent 62.2 ml, [AIBN] = 2wt.% relative to sum of 4-VP and EDMA together, temperature 70 °C, kaempferol = 10% (mole fraction) relative to 4-VP.

As a control experiment, non-imprinted polymer (NIP) microspheres were also prepared in identical manner but without the addition of kaempferol.

### 2.3. Physical characterization techniques

The surface morphology of MIP microsphere was observed by scanning electron microscopy (S-3400N, HITACHT, Japan). The particle size distributions of the MIP microspheres were measured using a laser diffraction particle size analyzer (Mastersizer 3000, Malvern Instruments Ltd, UK). Porosity and surface area analyses were performed by nitrogen sorption porosimetry (ASAP 2020M, Micromeritics Micromeritics Instrument Ltd, USA).

### 2.4. Adsorption properties

The adsorption properties of MIP microspheres were performed in 50 mL conical flask undergoing shaking (180 rpm) in a rocking table at 25 °C. For each adsorption experiment, 50.0 mg MIP microspheres was added in kaempferol/ethanol solution (10 mL) and mixed completely. After adsorption, the MIP microspheres were separated by organic ultrafiltration membrane (average pore size is 0.45 μm) and the concentration of the kaempferol in the solution after adsorption was determined using the UV–vis spectrophotometer at a wavelength of 367 nm. The adsorption capacity (Q) was calculated as follow: $Q=\left(C_{0}-C_{t}\right)\cdot V/W$, Where *C*_0_ and *C*_*t*_ is the kaempferol concentration (mg L^−1^) at initial and *T* time; *V* is the volume of solution (L), and *W* is the weight of dry MIP microspheres (g).

The adsorption kinetic and adsorption isotherm were used to evaluate the adsorption capacity of the optimal MIP microspheres to kaempferol. In the adsorption kinetic part, the kaempferol concentration of initial feed was 100 mg L^−1^, the temperature was 25 °C and the adsorption time was ranged from 0 to 180 min. In the adsorption isotherm part, the adsorption time was 120 min, the temperature was 25 °C and the kaempferol concentration was ranged from 20 to 180 mg L^−1^.

### 2.5. Selectivity experiment

Quercetin was selected as an interfering substance to evaluate the selectivity of MIP to kaempferol. When quercetin was used as interfering substance, the MIP microspheres were placed in a 10 ml of ethanol solution containing quercetin and kaempferol (the initial concentration of quercetin and kaempferol were both 50 mg L^−1^). The concentration of quercetin and kaempferol in the mixture solution were measured by the double-peak dual-wavelength method [22] using the UV–vis spectrophotometer. The maximum absorption wavelengths of kaempferol and quercetin were 367 and 373 nm, respectively, after further work, the concentration of kaempferol and quercetin in the mixed solution were measured by the simultaneous Equation (1) as following:

(1) $$\left\{\begin{array}{c} A_{367}=0.0685C_{\text{kaempferol}}+0.0830C_{\text{quercetin}}\\ A_{373}=0.0670C_{\text{kaempferol}}+0.0856C_{\text{quercetin}} \end{array}\right.$$

The selectivity of the MIPs to kaempferol was evaluated by separation factor (*α*) that was calculated with *α* = *K*_Di_/*K*_Dj_, where *K*_Di_ and *K*_Dj_ (mL g^−1^) were equilibrium dissociation constant of template molecule and interfering molecule respectively. The equilibrium dissociation constant *K*_D_ was calculated with *K*_D_ = *Q*_e_/*C*_e_, where *Q*_e_ (mg g^−1^) and *C*_e_ (mg mL^−1^) were equilibrium adsorption capacity of the MIPs and equilibrium concentration of feed solution.

## 3. Results and discussion

### 3.1. Synthesis of monodisperse MIP microspheres

#### 3.1.1. Influence of co-monomers’ concentration

**Figure 1.**
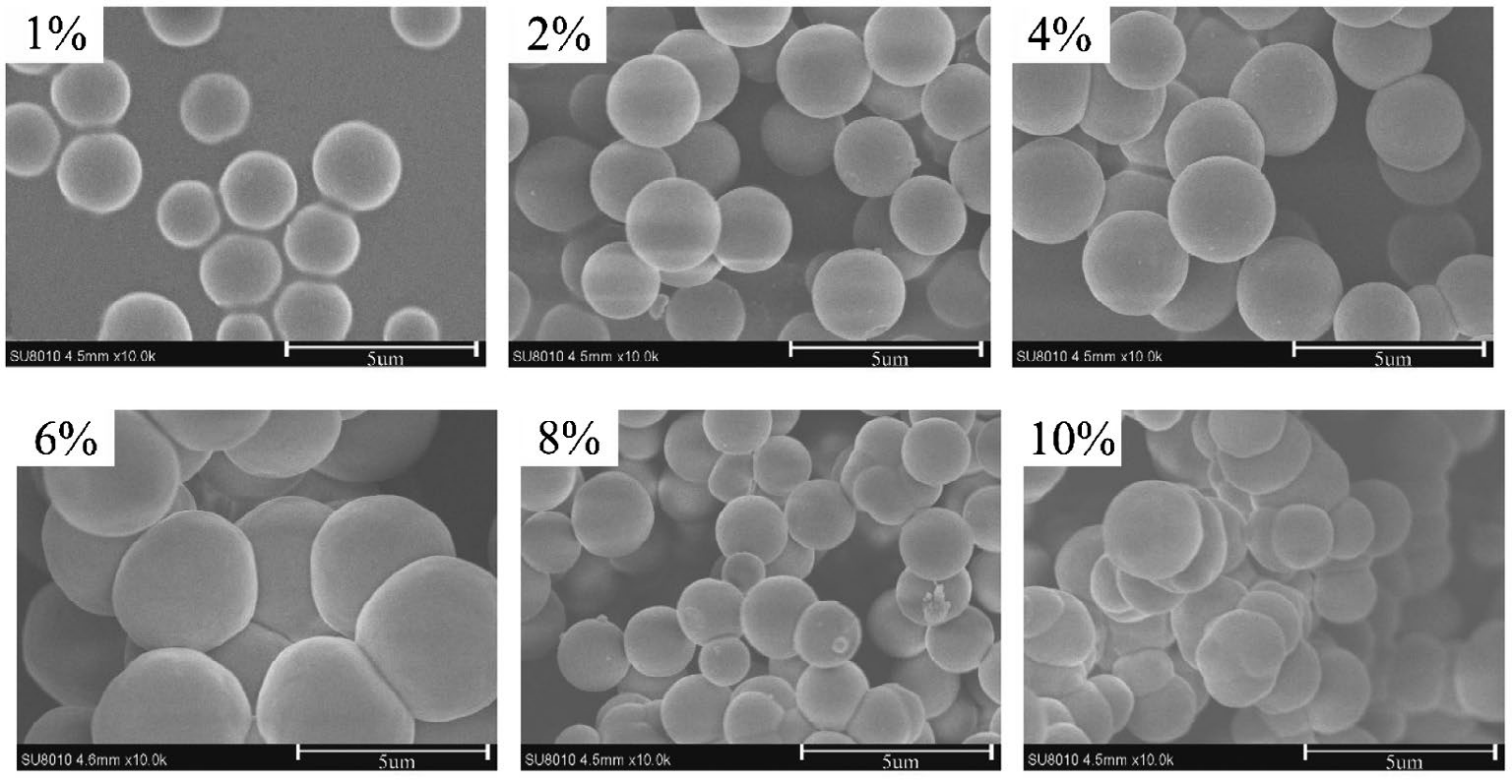
SEM micrographs of MIP particles at different co-monomers’ concentration.

As we known, proper concentration of co-monomers is very important to form monodisperse MIP microspheres, which is because of that there is no enough space for particles growth independently to form monodisperse MIP microspheres at high concentration of co-monomers. Figure 1 shows the particle morphology of the kaempferol MIP prepared from 1 to 10% (w/v) co-monomers in acetonitrile. Monodisperse MIP microspheres were isolated only from the polymerizations containing 1 and 2% (w/v) co-monomer. At the concentration of 4, 6, and 8% (w/v) co-monomers in acetonitrile, the MIPs still could form spherical particles, but the spherical particles agglomerated together. Only MIP coagulum could be obtained when concentration of co-monomers is 10% (w/v).These results demonstrate that 4-VP and EDMA can be polymerized to form monodisperse MIP microspheres if the co-monomers’ concentration is proper even without any stabilizer. MIP microspheres synthesis predominantly occurs by polymerization of 4-VP and EDMA, highly cross linked of EDMA formed rigid surfaces, which can prevent the growing MIP microspheres agglomeration, so nearly monodisperse and spherical particles can be routinely formed. At higher co-monomers’ concentration, amount of primary particles were formed firstly, however, the space was not enough for the growth for small particles independently, and particles would be connected together by oligomer, which lead to the agglomeration of MIP particles. In addition, when co-monomers’ concentration beyond 2%, the collision frequencies of the particles would increase because of the higher solid loadings, which also lead to the agglomeration of MIP microspheres.

**Figure 2.**
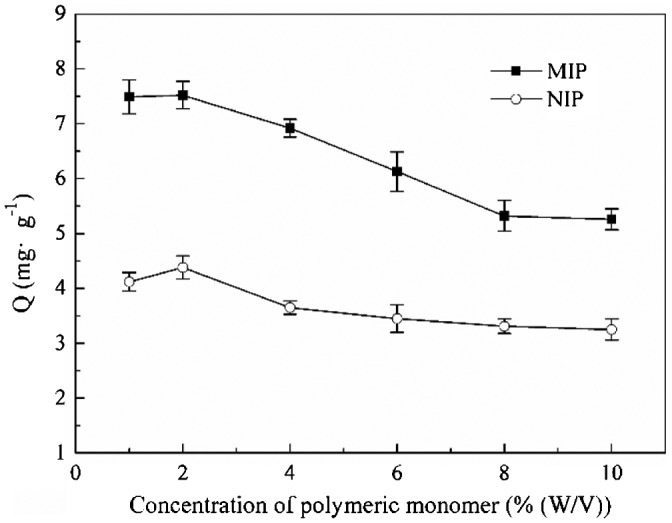
Adsorption capacity of MIP and NIP particles at different co-monomers’ concentration.

It can be seen from Figure 2 that the adsorption capacity of MIP microspheres was higher than that of NIP microspheres, which was the result of imprinting sites in the MIP microspheres instead of NIP microsphere. Figure 2 also shows that when co-monomers’ concentration increased from 1 to 2%, the adsorption capacity of the MIP had no remarkable change is almost the same, but after 2%, the adsorption capacity of MIP was decreased gradually with the decline of co-monomers’ concentration, and when the co-monomers’ concentration increased to 8%, the adsorption capacity had no remarkable change with the further rise of co-monomers’ concentration. The adsorption capacity of MIP was related with particles morphology, the monodisperse MIP microspheres had bigger specific surface area than coagulum, so more imprinting sites were presented in the surface of MIP microspheres, which accounted to a higher adsorption of MIP microspheres. When the co-monomers’ concentration increased from 2 to 8%, the sizes of the MIP microspheres became bigger (shown in Figure 1), so the specific surface area decreased, which led to a decline of adsorption capacity. Besides, the agglomeration became heavier with the increase of co-monomers’ concentration from 2 to 8%, which probably destroy some imprinting sites, and result in a decline of adsorption capacity. After the co-monomers’ concentration increased to 8%, the morphology of the MIP was always coagulum with the further increase of co-monomers’ concentration, so the adsorption capacity was no more change with further increase of co-monomers’ concentration after 8%. Considering the morphology and adsorption, 2%（w/v）was selected as the optimal concentration of co-monomers, and was kept in the following experiment.

#### 3.1.2. Molar ratios of 4-VP/EDMA

**Figure 3.**
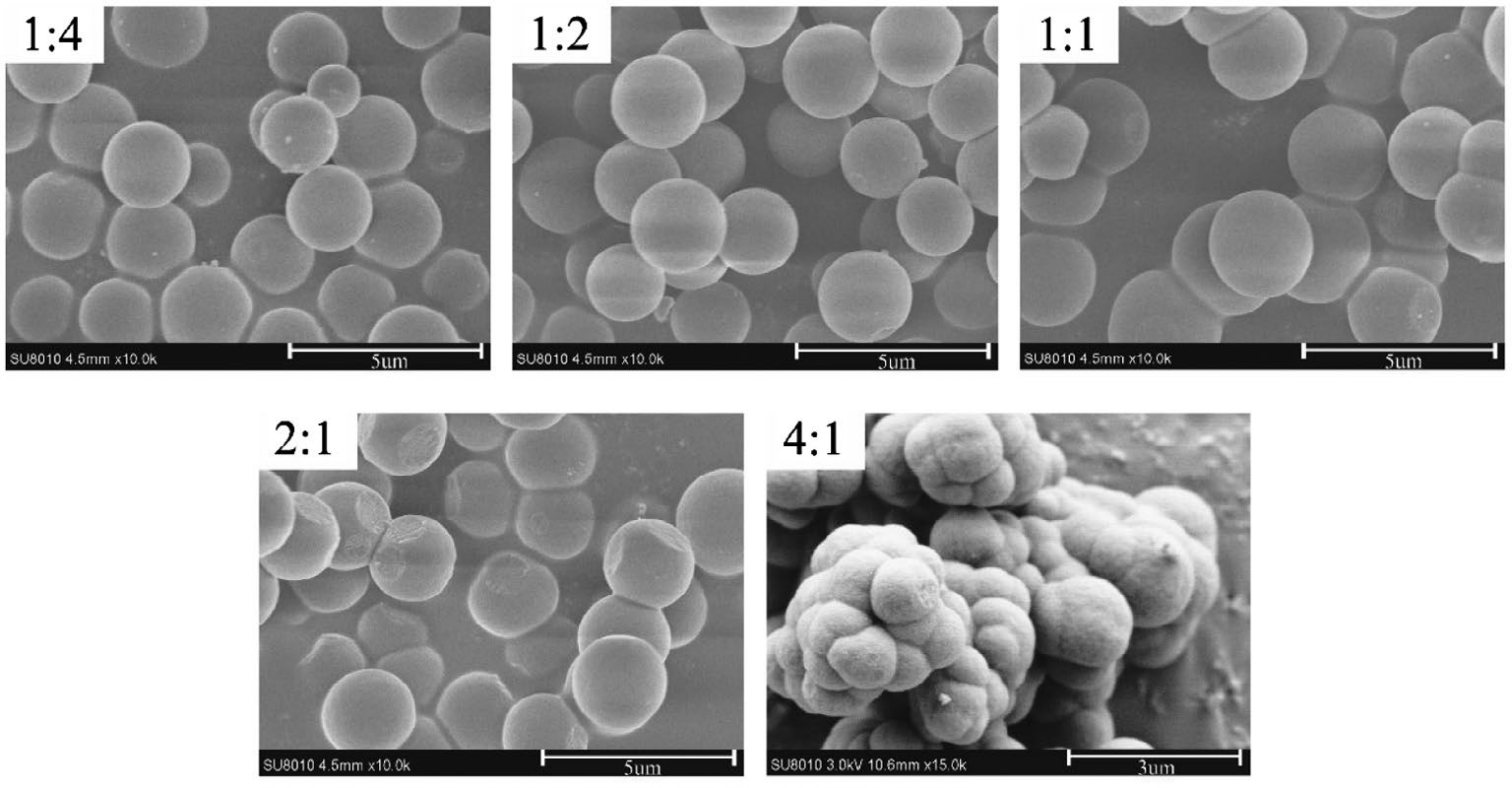
SEM micrographs of MIP particles at different molar ratios of 4-VP/EDMA.

A high percentage of cross-linker in the co-monomers mixture played an important role in controlling the morphology and binding characters of the achieved MIP microspheres. Figure 3 shows the particle morphology of MIP with the different molar ratios of 4-VP/EDMA, monodisperse MIP microspheres can be formed in the polymerizations which molar ratios were 1:4 and 1:2, but when the molar ratios change to 1:1, 2:1, and 4:1, the MIP microspheres become to agglomeration, and with the decrease of EDMA, the agglomeration was heavier, which indicate that a high crosslink degree is essential to prevent particle agglomeration. It has been reported that cross-linkers can form a rigid surface of the primary particles, which makes it difficult to agglomeration.[21,23] At lower percentage of cross-linker, the microspheres will be more easily swollen by the monomer and solvent, making the particles stickier which facilitate MIP microspheres more easy to agglomeration.

**Figure 4.**
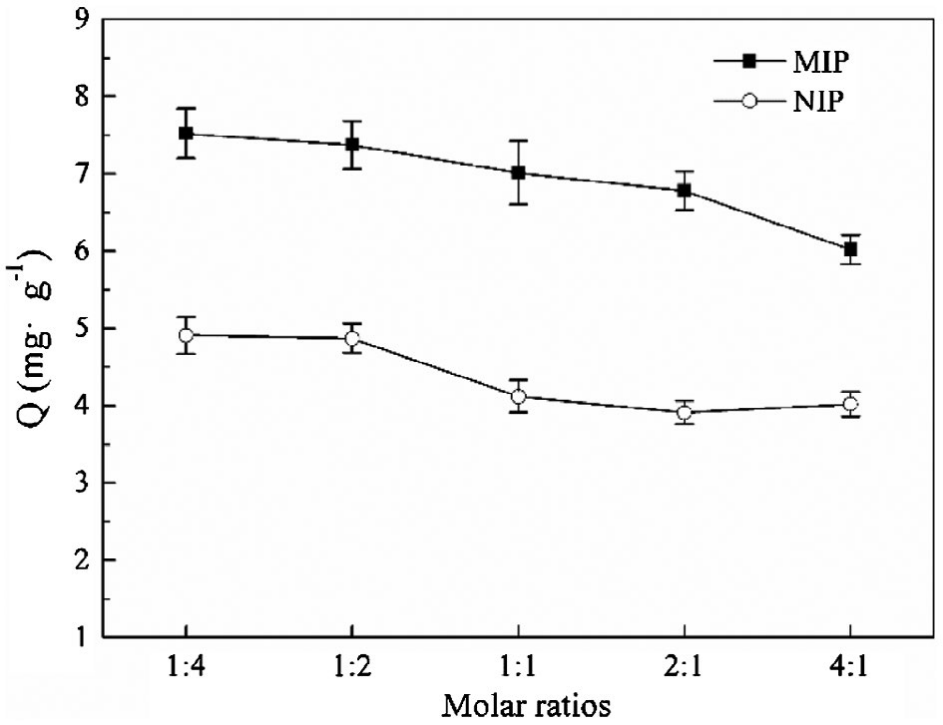
Adsorption capacity of MIP particles at different molar ratios of 4-VP/EDMA.

It can be seen from Figure 4 that with the decrease of cross-linker, the adsorption capacity decreased slightly, which also can be explained by the change of MIP’s morphology. The decline of cross-linker led to agglomeration of MIP particles, which result in a decrease of specific surface area and the number of effective imprinting sites, so the adsorption capacity decreased gradually. By analyzing the above result, 1:4 was considered as the optimal ratio of 4-VP and EDMA.

#### 3.1.3. Influence of polymerization time

**Figure 5.**
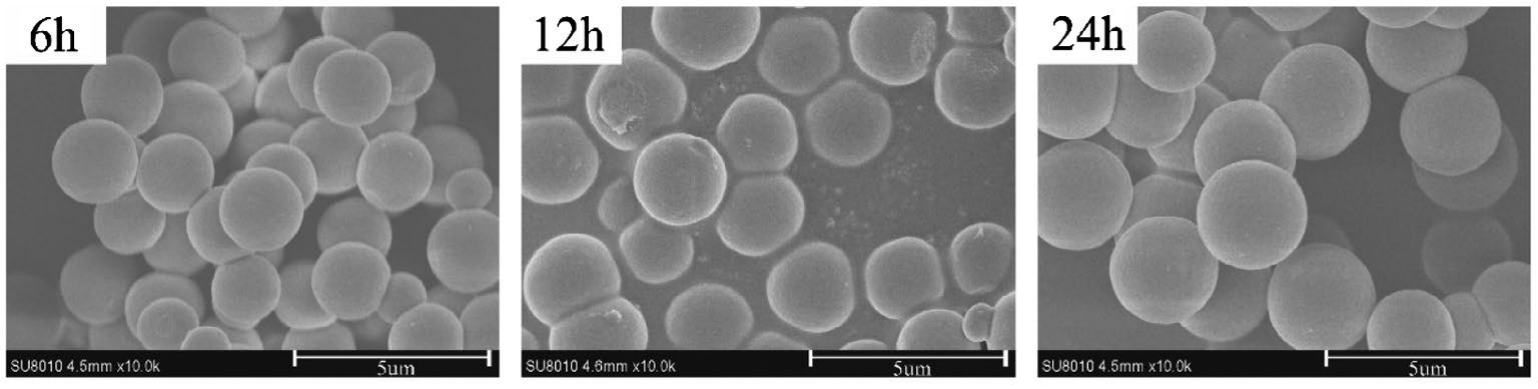
SEM micrographs of MIP particles at different polymerization time.

**Figure 6.**
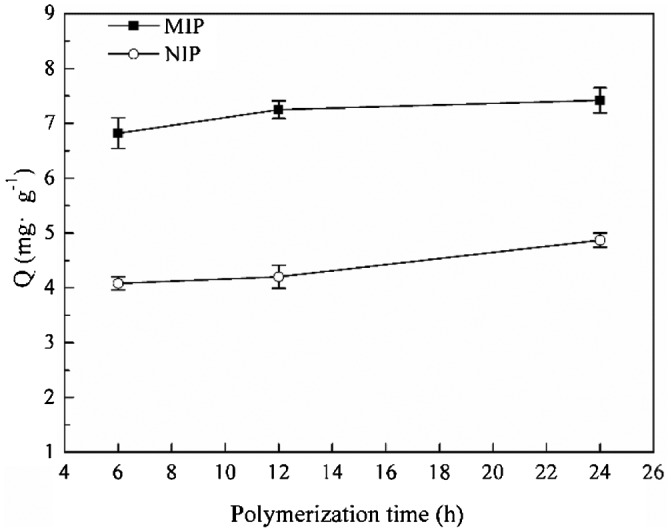
Adsorption capacity of MIP particles at different polymerization time.

Figure 5 shows the influence of polymerization time on the MIPs’ morphology. It can be found that the agglomeration of MIP microspheres were more heavily at 24 h than that of 6 and 12 h. Additional, with the increase of polymerization time, the average diameter of MIP microspheres increased slightly. In the early time of polymerization, particles with small diameters have enough space to grow up; with the increase of particles’ diameters, the space in the solution isn’t enough, and particles became agglomeration more heavily. Although the MIP microspheres that formed through 6 h polymerization can keep regular sphere morphology, but the adsorption capacity was slightly lower than the MIP microspheres that polymerization for 24 h (shown in Figure 6). This may be because that at 6 h, the MIP microspheres was not stable enough to maintain the structure of imprinting cavities. So the polymerization time was selected as 24 h to keep the stable structure of MIP microspheres.

### 3.2. Physical characterization of optimal MIP and NIP microspheres

In this work, by comparing of particle morphology and adsorption capacity of the MIP microspheres from different conditions, an optimal polymerization condition was obtained and is given in the experimental section. Although it is the imprinted polymer that is responsible for the selectivity separation of the target molecule, the physical properties of also contribute to the separation characteristics. Thus, the diameter distribution, pore volume, man pore size, and surface area of the optimal MIP microspheres were investigated. In addition, the physical characteristics of corresponding NIP microspheres were also measured to compare with MIP microspheres.

**Figure 7.**
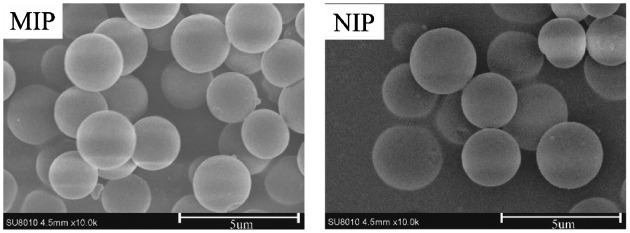
SEM micrographs of the optimal MIP and NIP microspheres.

**Figure 8.**
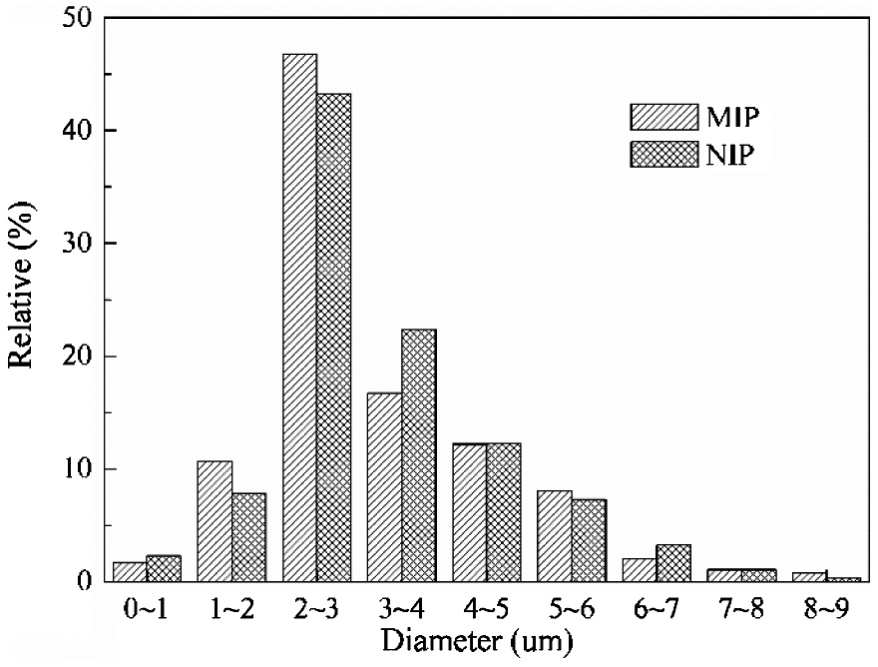
Particle size distribution of the optimal MIP microspheres.

Figure 8 shows the particle size distribution of optimal MIP and NIP microspheres, the diameter of both MIP and NIP microspheres were mainly 2–3 μm, which were corresponding with the particles’ diameters that measured from SEM micrographs in the Figure 7. Only fewer particle diameters bigger than 6 μm or smaller than 1 μm, but on the whole, sizes of the optimal MIP microspheres were in narrow disperse. Table 2 shows the pores properties of the optimal MIP and NIP microspheres, it can be concluded that the synthesized MIP and NIP microspheres were the microporous material and the pores were manly formed by the porogenic solvent acetonitrile.

**Table 2.** Pore properties of the optimal MIP and NIP microspheres.

	Pore volume (mL g^−1^)	Mean pore-size (Å)	BET- surface area (m^2^ g^−1^)
MIP	0.15	83.9	34.6
NIP	0.18	81.5	36.2

By comparing the SEM micrographs (Figure 7), the diameter distribution (Figure 8), pore volume, man pore size and surface area (Table 2) of the optimal MIP and NIP microspheres, it can be found that the particle morphology and physical characteristics of NIP were similar with corresponding MIP microspheres. It demonstrated that the presence of template molecule in the polymerization did not influence significantly the particle morphology and physical characteristics.

### 3.3. Adsorption studies of the optimal MIP microspheres

#### 3.3.1. Kinetic adsorption

**Figure 9.**
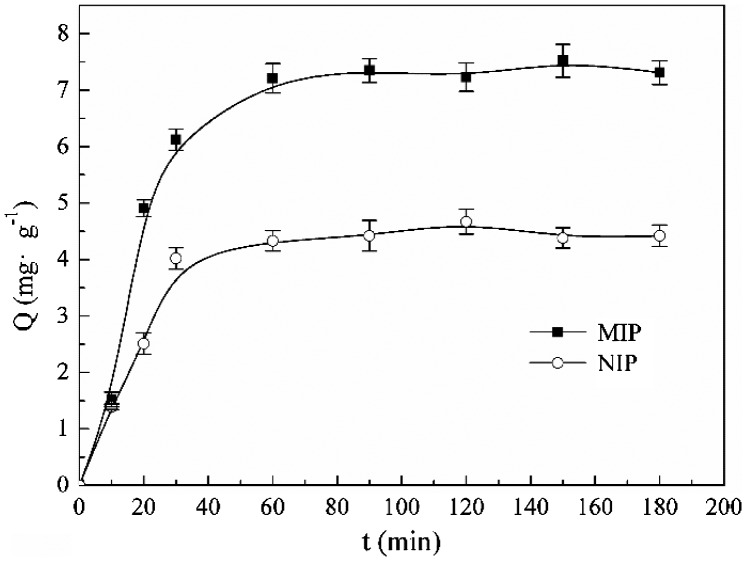
Kinetic adsorption curve of the optimal MIP and NIP microspheres.

The kinetic adsorption curves of spherical MIP and NIP are shown in Figure 9. Only after 30 min, the adsorption amount of the MIP was up to 6.12 mg g^−1^, which is 80% of the equilibrium adsorption capacity. As the adsorption prolonged, the adsorption amount increased slowly, after 60 min, the adsorption of MIP microspheres towards kaempferol reached to equilibrium, and the equilibrium adsorption was 7.35 mg g^−1^. The adsorption kinetic of NIP nanoparticles was also examined, the adsorption capacity kept low as the adsorption time prolonged, which is because no imprinted sites in the NIP. The adsorption of the NIP was the result from the huge specific surface area.

In order to further analyze the adsorption of MIP microspheres to kaempferol, the pseudo-first-order Equation (2) and pseudo-second-order Equation (3) were given as the following:

(2) $$\mathrm{lg}\left(Q_{\text{e}}-Q_{\text{t}}\right)\;\text{=}\;\mathrm{lg}Q_{\text{e}}-\frac{K_{1}}{2.203}t$$

(3) $$\frac{t}{Q_{\text{t}}}\;\text{=}\;\frac{1}{K_{2}Q_{\text{e}}^{2}}\;\text{+}\;\frac{t}{Q_{\text{e}}}$$

Where *Q*_e_ and *Q*_t_ (mg g^−1^) were the amount of adsorption at the equilibrium and *t* time, respectively. *K*_1_ (min^−1^) and *K*_2_ (g mg^−1^ min^−1^) were the adsorption rate constant of pseudo-first-order and pseudo-second-order, respectively. The value of *K*_1_ was calculated from the plots of lg(*Q*_e_−*Q*_t_) vs. *t* by pseudo-first-order, and *K*_2_ was obtained from plotting (*t Q*_t_^−1^) vs. *t* by the pseudo-second-order.

By comparing *R* ^2^ of Pseudo-first-order kinetics and Pseudo-second-order kinetics (Table 3), the adsorption kinetic of optimal MIP microspheres was fitted for the pseudo-second-order kinetic model better, it can be concluded that the adsorption of MIP microsphere to kaempferol was dominated by chemical adsorption, and such intermolecular force between imprinted site and kaempferol was mainly hydrogen-bridge bond. However, the adsorption of MIP was not simply hydrogen-bridge bond, there were still some other physical interactions, for example Van der Waals force, contribute to the binding of imprinted sites and kaempferol.

**Table 3.** Parameters of pseudo-first-order and pseudo-second-order kinetic models of the optimal MIP microspheres.

Pseudo-first-order kinetic parameters		Pseudo-second-order kinetic parameters	
*K*_1_ (min^−1^)	*R*^2^	*K*_2_ (g mg^−1^ min^−1^)	*R*^2^
0.0228	0.7783	0.0072	0.9663

#### 3.3.2. Adsorption isotherm

**Figure 10.**
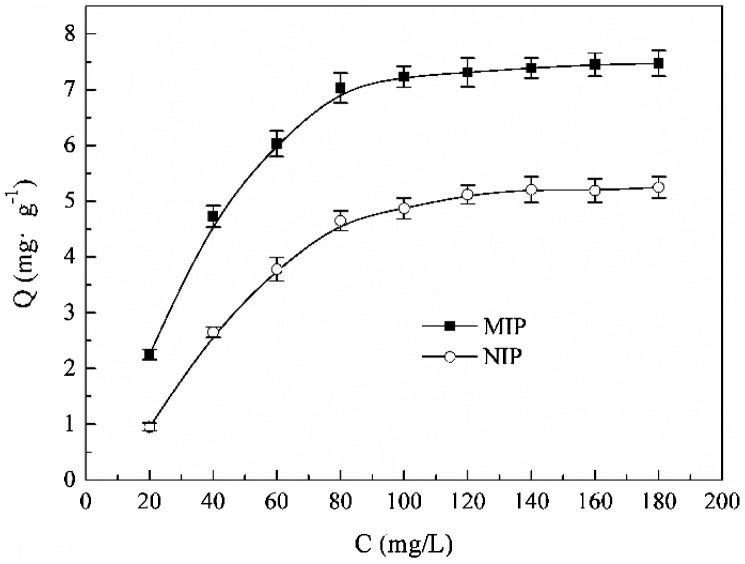
Adsorption isotherms of the optimal MIP and NIP microspheres.

From the adsorption isotherms data of MIP and NIP microspheres for kaempferol shown as Figure 10, it can be found that the adsorption capacity all increased with rise of kaempferol concentration. This increase trend was more obvious firstly, and after the kaempferol concentration increase to 60 mg L^−1^, the adsorption capacity tends to equilibrium. When the kaempferol concentration increased from 20 to 100 mg L^−1^, more kaempferol molecules would be presented in the solution to be adsorbed by the MIP, so the adsorption capacity increased gradually. But the imprinted sites in the MIP would be occupied completely after the kaempferol concentration exceed 60 mg/L, and the excess kaempferol won’t be adsorbed any more. The saturated adsorption capacity of spherical MIPs is 7.47 mg g^−1^, and the adsorption capacity of NIP was lower than MIP for the same reason that analyzed as kinetic adsorption part.

For further investigating the adsorption of MIP microspheres, Langmuir isothermal Equation (4) and the Freundlich isothermal Equation (5) were used to measure the equilibrium adsorption, respectively:

(4) $$\frac{C_{e}}{Q_{e}}=\frac{1}{Q_{\max}}C_{e}+\frac{1}{Q_{\max}K_{\text{L}}}$$

(5) $$\mathrm{lg}Q_{e}=\mathrm{lg}K_{\text{f}}+\frac{1}{n}\mathrm{lg}C_{e}$$

Where *Q*_e_ (mg g^−1^) and *C*_e_ (mg L^−1^) were the amount adsorption and the concentration of residual kaempferol in the solution at equilibrium respectively, *Q*_max_ (mg g^−1^) is the theoretical maximum adsorption capacity calculated by Langmuir adsorption equation, *K*_L_ (L mg^−1^) and *K*_f_ were the Langmuir constant and Freundlich constants respectively. The n was the coefficient that describes the variation trend of adsorption isothermal.

It can be seen from the Table 4 that the adsorption isotherm can be well fitted with Langmuir isothermal models rather than Freundlich parameters, which means that the adsorption of MIP microspheres towards kaempferol was single layer adsorption. The adsorption was mainly lies in the imprinting sites of MIP microspheres, when these imprinting sites were occupied, residual kaempferol couldn’t be adsorbed by the MIP microspheres.

**Table 4.** Parameters of Langmuir isothermal and Freundlich isothermal of the optimal MIP microspheres.

Langmuir parameters			Freundlich parameters		
*Q*_max_ (mg g^−1^)	*K*_L_ (L mg^−1^)	*R*^2^	*K*_f_	*n*	*R*^2^
8.38	0.0719	0.9911	1.421	2.73	0.7801

#### 3.3.3. Selectivity experiment

Separation factor (*α*) was an important factor to evaluate the selectivity of spherical MIP, which can be calculated by the equilibrium dissociation constant (*K*_D_). Table 5 shows the equilibrium dissociation constant (*K*_D_) of MIP and NIP microspheres to kaempferol and quercetin, respectively, as well as the calculated separation factors (*α*). Obviously, MIP hold a higher selectivity to kaempferol in the present of competitor quercetin, which separation factor was up to 3.91, but NIP show no selectivity to kaempferol. The imprinted sites in the MIP were designed for kaempferol, and only bind with kaempferol, which accounts to a high selectivity to kaempferol. However, from Table 5, it can be seen that the equilibrium dissociation constant of MIP microspheres to quercetin was 19.25, which indicate that MIP microspheres also adsorbed a little amount of quercetin, this part of adsorption was the result from huge specific surface area of MIP microspheres. NIP microspheres had no imprinted sites, its adsorption to kaempferol and quercetin were also the result from the huge specific surface area of NIP microspheres.

**Table 5.** Equilibrium dissociation constant (*K*_D_) and separation factor (*α*) of optimal MIP microspheres towards kaempferol and quercetin.

Adsorbent	Target	*K*_D_	*α*
MIP	Kaempferol	75.27	3.91
	Quercetin	19.25	
NIP	Kaempferol	18.52	1.01
	Quercetin	18.31	

## 4. Conclusion

In summary, we have successfully prepared monodisperse MIP microspheres by precipitation polymerization using 4-VP as functional monomer and EDMA as cross-linker. The new MIP microspheres offer the uniform spherical shape, which work-up procedure was simplified and without any stabilizers or emulsifiers. The sizes of optimal MIP microspheres were mainly disperse in 2–3 μm, and the pores properties demonstrated that the optimal microspheres were microporous material. The adsorption capacity of was relative with the particles’ morphology, monodisperse MIP microspheres’ adsorption capacity were better than agglomeration. The evaluation of optimal MIP microspheres’ adsorption indicated that MIP microspheres exhibit a higher adsorption capacity and selectivity to kaempferol, the saturated adsorption capacity was 7.47 mg g^−1^, and the adsorption equilibrium could be obtained in 30 min. The adsorption of the MIP microspheres was better fit pseudo-second-order kinetic and Langmuir isothermal models, which illustrate that the adsorption mechanism were chemical adsorption and single layer adsorption. More importantly, the separation factor was up to 3.91 with the present of quercetin, which indicated that the synthesized MIP microspheres had a high selectivity to kaempferol. So the MIP microspheres were potential in the kaempferol separation.

## Disclosure statement

No potential conflict of interest was reported by the authors.

## Funding

This work was supported by the National Science Foundation of China [grant number 21376030] and the Fundamental Research Funds for the Central Universities [grant number 2015ZCQ-LY-03].
